# Anti-adult T-cell leukemia/lymphoma effects of indole-3-carbinol

**DOI:** 10.1186/1742-4690-6-7

**Published:** 2009-01-16

**Authors:** Yoshiaki Machijima, Chie Ishikawa, Shigeki Sawada, Taeko Okudaira, Jun-nosuke Uchihara, Yuetsu Tanaka, Naoya Taira, Naoki Mori

**Affiliations:** 1 Division of Molecular Virology and Oncology, Graduate School of Medicine, University of the Ryukyus, 207 Uehara, Nishihara, Okinawa, Japan; 2 Division of Child Health and Welfare, Faculty of Medicine, University of the Ryukyus, 207 Uehara, Nishihara, Okinawa, Japan; 3 The Japanese Society for the Promotion of Science (JSPS), Japan; 4 Division of Oral and Maxillofacial Functional Rehabilitation, Faculty of Medicine, University of the Ryukyus, 207 Uehara, Nishihara, Okinawa, Japan; 5 Division of Endocrinology and Metabolism, Faculty of Medicine, University of the Ryukyus, 207 Uehara, Nishihara, Okinawa, Japan; 6 Depertment of Internal Medicine, Naha City Hospital, 2-31-1 Furujima, Naha, Okinawa, Japan; 7 Division of Immunology, Faculty of Medicine, University of the Ryukyus, 207 Uehara, Nishihara, Okinawa, Japan; 8 Department of Internal Medicine, Heartlife Hospital, 208 Iju, Nakagusuku, Okinawa, Japan

## Abstract

**Background:**

Adult T-cell leukemia/lymphoma (ATLL) is a malignancy derived from T cells infected with human T-cell leukemia virus type 1 (HTLV-1), and it is known to be resistant to standard anticancer therapies. Indole-3-carbinol (I3C), a naturally occurring component of *Brassica *vegetables such as cabbage, broccoli and Brussels sprout, is a promising chemopreventive agent as it is reported to possess antimutagenic, antitumorigenic and antiestrogenic properties in experimental studies. The aim of this study was to determine the potential anti-ATLL effects of I3C both *in vitro *and *in vivo*.

**Results:**

In the *in vitro *study, I3C inhibited cell viability of HTLV-1-infected T-cell lines and ATLL cells in a dose-dependent manner. Importantly, I3C did not exert any inhibitory effect on uninfected T-cell lines and normal peripheral blood mononuclear cells. I3C prevented the G_1_/S transition by reducing the expression of cyclin D1, cyclin D2, Cdk4 and Cdk6, and induced apoptosis by reducing the expression of XIAP, survivin and Bcl-2, and by upregulating the expression of Bak. The induced apoptosis was associated with activation of caspase-3, -8 and -9, and poly(ADP-ribose) polymerase cleavage. I3C also suppressed IκBα phosphorylation and JunD expression, resulting in inactivation of NF-κB and AP-1. Inoculation of HTLV-1-infected T cells in mice with severe combined immunodeficiency resulted in tumor growth. The latter was inhibited by treatment with I3C (50 mg/kg/day orally), but not the vehicle control.

**Conclusion:**

Our preclinical data suggest that I3C could be potentially a useful chemotherapeutic agent for patients with ATLL.

## Background

Adult T-cell leukemia/lymphoma (ATLL) is a fatal T-cell malignancy caused by infection of mature CD4^+ ^T cells by human T-cell leukemia virus type 1 (HTLV-1) [1-3]. ATLL is clinically and hematologically subclassified into four subtypes: acute, lymphoma, chronic and smoldering. In the relatively indolent smoldering and chronic types, the median survival time is ≥ 2 years. However, currently, there is no accepted curative therapy for ATLL and the condition often progresses to death with a median survival time of 13 months in aggressive ATLL [4]. Death is usually due to severe infection or hypercalcemia, often associated with resistance to intensive, combined chemotherapy. Therefore, the establishment of new therapeutic strategies for ATLL is deemed critical.

ATLL arises after a long latent period of over 50 years and involves a multi-step mechanism of tumorigenesis [5]. Although the mechanism of transformation and leukemogenesis is not fully elucidated, there is evidence to suggest that the viral oncoprotein Tax plays a crucial role in these processes through the regulation of several pathways including NF-κB and the cell-cycle pathways [6-8]. The observation that Tax-induced NF-κB is indispensable for the maintenance of the malignant phenotype of HTLV-1, through the regulation of expression of various genes involved in cell-cycle regulation and inhibition of apoptosis, provides a possible molecular target for ATLL.

Indol-3-carbinol (I3C) is an autolysis product of a glucosinolate, glucobrassicin, found in *Brassica *species or cruciferous vegetables such as cabbage, broccoli, cauliflower and Brussels spouts [9,10]. The chemopreventive potential of I3C has received much attention in light of its reported *in vivo *efficacy in protection against chemically-induced carcinogenesis in animals [11-13]. Moreover, the clinical benefits of I3C have also been shown in human clinical trials for cervical dysplasia [14], breast cancer [15,16] and vulvar intraepithelial neoplasia [17]. Despite these advances in translational research, the mechanism by which I3C inhibits tumorigenesis remains inconclusive. Mechanistic evidence indicates that I3C facilitates growth arrest and apoptosis by targeting a broad range of signaling pathways pertinent to cell-cycle regulation and survival, including those mediated by Akt, NF-κB and mitogen-activated protein kinases [18-21]. However, as these signaling targets often operate in a cell-specific fashion, it remains controversial whether any of them solely accounts for the effect of I3C on growth arrest and apoptosis of tumor cells [22].

I3C has also been shown to suppress the proliferation of various tumor cells including breast cancer, prostate cancer, endometrial cancer, colon cancer and myeloid leukemia cells [21]. However, the potential of I3C to inhibit the proliferation of ATLL cells has not been evaluated. In this study, we investigated the effects of I3C on cell growth and apoptosis of HTLV-1-infected and uninfected T-cell lines and primary ATLL cells. The results demonstrated selective effects on HTLV-1-infected malignant T cells and support a potential therapeutic role for I3C in patients with ATLL.

## Results

### I3C inhibits cell viability of HTLV-1-infected T-cell linesand primary ATLL cells

**Figure 1:**
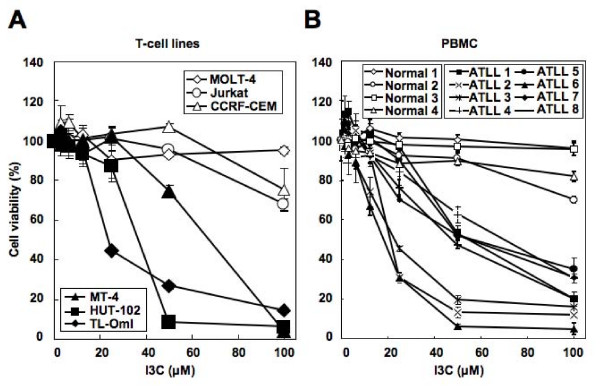
**Inhibitory effects of I3C on cell viability of HTLV-1-infected T-cell lines and primary ATLL cells**. Cell lines and PBMC were incubated in the presence of various concentrations of I3C for 72 h and 24 h, respectively, and viability of the cultured cells was measured by WST-8 assay. Relative viability of cultured cells is presented as the mean determined on cell lines (A) and PBMC from healthy controls and ATLL patients (B) from triplicate cultures. A relative viability of 100% was designated as total number of cells that grew in 72-h cultures in the absence of I3C. HUT-102, MT-4 and TL-OmI are HTLV-1-infected T-cell lines; Jurkat, MOLT-4 and CCRF-CEM, uninfected T-cell lines used as controls. Data are mean ± SD of triplicate experiments.

First, we examined the effects of I3C on cell viability of HTLV-1-infected T-cell lines. We used two HTLV-1-transformed T-cell lines (MT-4 and HUT-102), an ATLL-derived T-cell line (TL-OmI) and three HTLV-1-negative T-cell lines (MOLT-4, Jurkat and CCRF-CEM). Tax protein was detected by immunoblot analysis in the two HTLV-1-transformed T-cell lines but not in the ATLL-derived T-cell line (data not shown). Cell viability was assessed by the water-soluble tetrazolium (WST)-8 assay kit. Culture of cells with various concentrations of I3C for 72 h resulted in the suppression of cell viability in a dose-dependent manner in all three lines (Figure 1A). The effect of I3C was not significant on control uninfected T-cell lines.

We also evaluated the effects of I3C on cell viability of fresh ATLL cells obtained from eight independent ATLL patients. As shown in Figure 1B, I3C inhibited cell viability of fresh ATLL cells. It seems that there are two groups of ATLL samples. One of them (ATLL 2, 3 and 6) is more sensitive to I3C than the other. However, there were no differences between two groups in terms of the clinical parameters, such as white blood cells count, proportion of ATLL cells, lactate dehydrogenase level and survival time (data not shown). All patients were negative for Tax protein by immunoblot analysis (data not shown). Importantly, I3C up to 100 μM had no effect on viability of normal peripheral blood mononuclear cells (PBMC) obtained from four healthy donors.

### I3C treatment causes G_1_/S cell-cycle arrest in HTLV-1-infected T-cell lines

**Figure 2:**
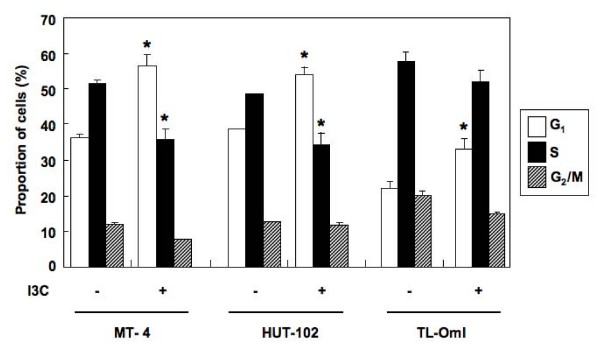
**I3C induces cell-cycle arrest in HTLV-1-infected T-cell lines**. HTLV-1-infected T-cell lines were incubated in the absence or presence of I3C (100 μM) for 12 h and then stained with propidium iodide, and analyzed for DNA content by flow cytometry. Three independent experiments per cell line were performed and results are presented as the mean percentage ± SD. *P < 0.05, compared with control.

Next, we examined the cellular DNA contents distribution by flow cytometric analysis following cell treatment. In all HTLV-1-infected T-cell lines, I3C induced significant changes in the cell-cycle distribution (Figure 2). Cultivation with I3C for 12 h increased the population of cells in the G_1 _phase, with a marked reduction of cells in the S phase. These changes were primarily the result of a G_1_/S cell-cycle arrest in HTLV-1-infected T-cell lines. At 24 h after treatment, the population of cells in the pre-G_0_/G_1 _region, regarded as apoptotic cells, was increased (data not shown).

### I3C induces apoptosis of HTLV-1-infected T-cell lines

**Figure 3:**
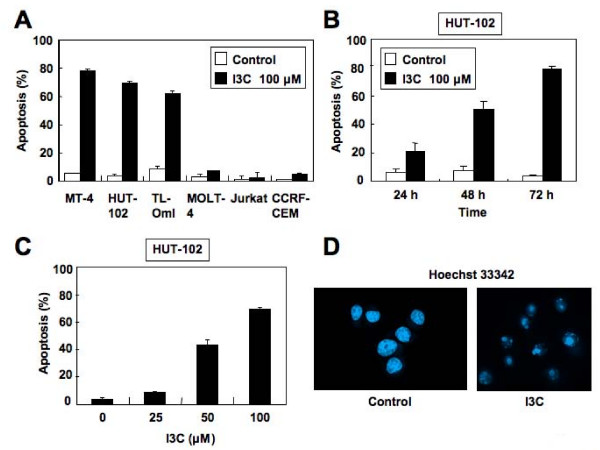
**I3C induces apoptosis of HTLV-1-infected T-cell lines**. (A) Cell lines were treated with or without I3C (100 μM) for 72 h. (B) I3C induces apoptosis of HUT-102 cells in a time-dependent manner. HUT-102 cells were treated with or without I3C (100 μM) for the indicated periods. (C) I3C induces apoptosis of HUT-102 cells in a dose-dependent manner. HUT-102 cells were treated with I3C at the indicated concentrations for 72 h. Cells were harvested, then stained with the APO2.7 monoclonal antibody and analyzed by flow cytometry. Data represent the mean percentage ± SD of apoptotic cells. (D) Hoechst 33342 staining. HUT-102 cells were treated with I3C (100 μM) for 48 h and stained by Hoechst 33342. Original magnification, × 1,000.

To check that the observed increase in the pre-G_0_/G_1 _results from apoptosis, I3C-treated HTLV-1-infected T-cell lines were analyzed by staining with APO2.7 monoclonal antibody. I3C increased the proportion of apoptotic cells in all HTLV-1-infected T-cell lines, but not in uninfected T-cell lines (Figure 3A). A significant increase in the apoptotic population was detected in HUT-102 cells in a time- and dose-dependent manner (Figure 3B and 3C). A similar experiment was also performed with Hoechst 33342 staining (Figure 3D). This staining allows evaluation of chromatin condensation, which is a hallmark of apoptosis. Consistent with the above results, I3C significantly increased DNA degradation in HUT-102 cells. Taken together, these results indicate that I3C inhibits cell viability of HTLV-1-infected T-cell lines through cell apoptosis.

### I3C-induced apoptosis is caspase-dependent

**Figure 4:**
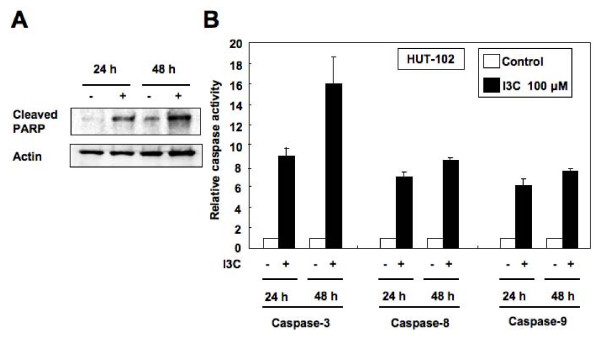
**I3C-induced apoptosis is caspase-dependent in HTLV-1-infected T-cell lines**. (A) Effect of I3C on the cleavage of PARP in HUT-102 cells. Cells were treated with I3C (100 μM) for the indicated periods. Total SDS protein lysates (20 μg per lane) were prepared and immunoblotted against cleaved PARP. Representative data of three experiments with similar results. (B) I3C treatment activates caspase-3, -8 and -9 in HUT-102 cells. Cells were treated with or without I3C (100 μM) for 48 h. Caspase activity was assayed as described in "Methods" and expressed relative to untreated cells, which were assigned a value of 1. Values represent the mean ± SD of three experiments.

We then investigated whether the observed apoptosis was due or not to caspase activation. Cell extracts were obtained after treatment and processed for Western blot. Indeed, in HUT-102 cells, I3C-induced apoptosis was associated with caspase activation, as shown by poly(ADP-ribose) polymerase (PARP) cleavage (Figure 4A). Furthermore, I3C treatment resulted in activation of caspases-3, -8 and -9 in HUT-102 cells (Figure 4B). These results demonstrate the involvement of caspase activation in I3C-induced apoptosis in HTLV-1-infected T-cell lines.

**Figure 5:**
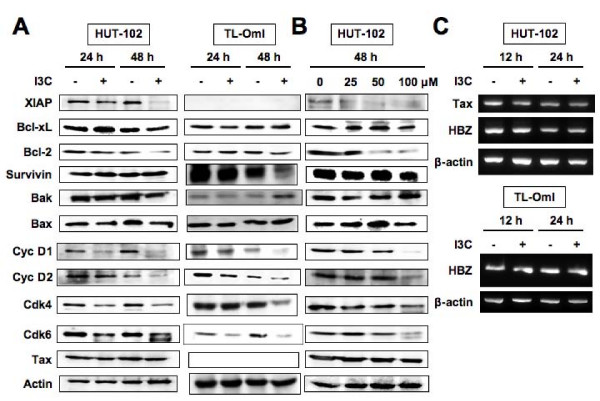
**Effects of I3C on the expression of cell-cycle and apoptosis regulatory proteins**. Western blot analysis of HUT-102 and TL-OmI cells treated with I3C. (A) Cells were treated with I3C (100 μM) for the indicated periods. (B) Cells were treated with I3C at the indicated concentrations for 48 h. Total cellular proteins (20 μg per lane) were separated on SDS-polyacrylamide gels and transferred to the membrane. Protein levels were detected by Western blotting with antibodies directed against each protein. (C) Total RNA was extracted from HUT-102 and TL-OmI cells following treatment with I3C (100 μM) for 12 or 24 h. The mRNA expression of Tax and HBZ was analyzed by RT-PCR analysis. β-actin served as an internal control in the RT-PCR procedure. Representative data of three experiments with similar results.

### Effects of I3C on cell-cycle and apoptosis regulatory proteins

To clarify the molecular mechanisms of I3C-induced inhibition of cell viability and apoptosis in HTLV-1-infected T-cell lines, we examined the expression of several intracellular regulators of cell-cycle and apoptosis, including cyclin D1, cyclin D2, Cdk4, Cdk6, Bak, Bax, Bcl-2, Bcl-xL, XIAP and survivin by Western blot analysis. As shown in Figure 5A, I3C did not alter Bax and Bcl-xL levels in HUT-102 and TL-OmI cells. In contrast, I3C significantly decreased the expression of cyclin D1, cyclin D2, Cdk4 and Cdk6 in HUT-102 and TL-OmI cells in a time-dependent manner. I3C dose-dependently decreased the levels of expression of these proteins (Figure 5B). Although XIAP was not detected in TL-OmI cells, I3C decreased its expression in HUT-102 cells in a time- and dose-dependent manner. I3C also decreased the expression of Bcl-2 and survivin in HUT-102 and TL-OmI cells, respectively. In contrast, the expression of Bak was increased in TL-OmI cells treated with I3C for 48 h. Western blot analysis showed that I3C treatment had no effect on Tax expression in HUT-102 cells (Figure 5A). Comparable loading of protein was confirmed with a specific antibody for the housekeeping gene product actin. Furthermore, mRNA expression of Tax and HBZ, which was recently identified in the 3'-long terminal repeat of the complementary sequence of HTLV-1 and has been suggested as a critical gene in leukemogenesis of ATLL [23], in HUT-102 and TL-OmI cells treated with I3C, was examined by RT-PCR. However, both mRNA expression levels were not affected by I3C treatment (Figure 5C).

### I3C modulates activated NF-κB and AP-1

**Figure 6:**
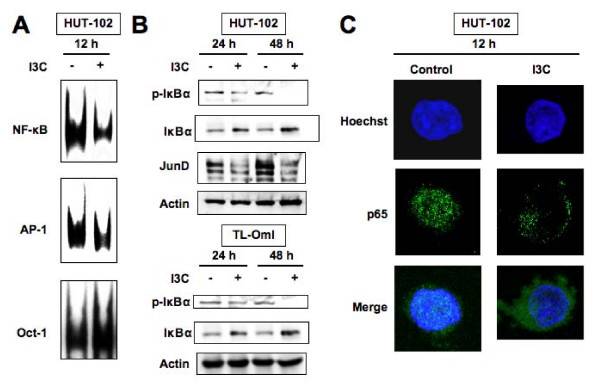
**I3C suppresses activities of nuclear NF-κB and AP-1 in HTLV-1-infected T-cell lines**. (A) Effects of 12-h treatment of HUT-102 cells with I3C (100 μM) on activation of NF-κB, AP-1 and Oct-1 assessed by EMSA using oligonucleotide probe for NF-κB, AP-1 or Oct-1. (B) Effects of I3C on the level of IκBα, phosphorylated IκBα (p-IκBα) and JunD by Western blot analysis. HUT-102 and TL-OmI cells were treated with I3C (100 μM) for the indicated periods, followed by protein extraction. Whole cell extracts (20 μg per lane) of treated cells were immunoblotted with specific antibodies. The arrowhead and arrow point to 38 kDa phosphorylated IκBα and 36 kDa IκBα protein, respectively. Representative data of three experiments with similar results. (C) Inhibition of nuclear translocation of NF-κB p65 by I3C. Representative results of immunofluorescence analyses in HUT-102 cells treated with I3C (100 μM) for 12 h using antibody against NF-κB p65. Original magnification, ×1,000.

NF-κB is a transcription factor involved in the control of apoptosis, cell-cycle progression and cell differentiation [24]. NF-κB is constitutively activated in Tax-expressing and HTLV-1-infected T-cell lines as well as primary ATLL cells [25], and such activation correlates with leukemogenesis [26]. Because NF-κB regulates the expression of cyclin D1, cyclin D2, Cdk4, Cdk6, Bcl-2, XIAP and survivin [27-32], we examined whether I3C inhibits the NF-κB pathway. To study the DNA-binding activity of NF-κB, we performed electrophoretic mobility shift assay (EMSA) with radiolabeled double-stranded NF-κB oligonucleotides and nuclear extracts from untreated or I3C-treated HUT-102 cells. NF-κB oligonucleotide probe with nuclear extracts from untreated HUT-102 cells generated DNA-protein gel shift complexes (Figure 6A, top panel). NF-κB complex contained p50, p65 and c-Rel [33]. Nuclear extracts prepared from HUT-102 cells treated with I3C for 12 h exhibited a decrease in the intensity of NF-κB-containing gel shift complexes (Figure 6A, top panel). This finding suggests that I3C downregulates the DNA-binding activities of NF-κB. Inhibition appeared specific to NF-κB and not due to cell death, because no significant change in binding activity of Oct-1 was observed after treatment of cells with I3C (Figure 6A, bottom panel).

Degradation of IκBα and subsequent release of NF-κB requires prior phosphorylation at Ser32 and Ser36 residues [34]. To investigate whether the inhibitory effect of I3C was mediated through alteration of phosphorylation of IκBα, HUT-102 and TL-OmI cells were treated with I3C and their protein extracts were checked for phospho-IκBα expression. Untreated HUT-102 and TL-OmI cells constitutively expressed Ser32/36-phosphorylated IκBα, while I3C treatment decreased the phosphorylated IκBα in a time-dependent manner (Figure 6B), with a concomitant rise in IκBα level. These results suggest that I3C inhibits phosphorylation of IκBα followed by accumulation of this protein. We next examined the effect of I3C on the cellular distribution of NF-κB components using fluorescence microscopy. I3C blocked nuclear localization of NF-κB p65 in HUT-102 cells, which constitutively express this protein in the cell nucleus in the absence of I3C (Figure 6C).

Transcription factor AP-1 is also identified as a crucial mediator of both cell-cycle enhancing and cell death inhibiting pathways in HTLV-1-infected T-cells [7]. Tax activates transcription through AP-1 site and induces AP-1 DNA-binding activity [35,36]. Therefore, we focused on AP-1 inactivation after exposure to I3C. HUT-102 cells exhibited elevated constitutive AP-1 DNA-binding activity (Figure 6A, middle panel). I3C also reduced AP-1 DNA-binding activity. HTLV-1-infected T-cells with increased AP-1 DNA-binding activity contained Jun D [35]. I3C decreased time-dependently the expression of JunD (Figure 6B). These findings suggest that I3C depletes JunD, resulting in inactivation of AP-1.

### Chemotherapeutic effects of I3C on subcutaneous HUT-102 tumors

**Figure 7:**
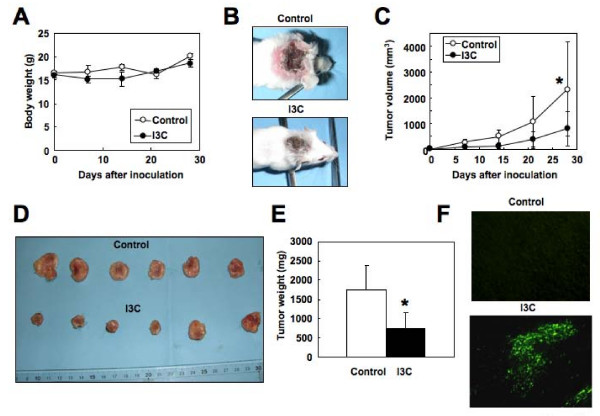
**I3C inhibits growth of HUT-102 cells in SCID mice**. HUT-102 cells (1 × 10^7 ^per mouse) were inoculated subcutaneously into SCID mice. The mice (*n *= 6/group) were treated with either vehicle or I3C (50 mg/kg given orally every day). Treatment was initiated on the day after inoculation. (A) Body weight of mice measured weekly for four weeks. (B) Photographs of an untreated mouse and I3C-treated mouse inoculated 28 days earlier with HUT-102 cells subcutaneously in the postauricular region. (C) The mice were monitored for tumor volumes at 7, 14, 21 and 28 days after cell inoculation. (D and E) Tumors removed from I3C-treated mice and untreated mice on day 28 after cell inoculation were weighed. *P < 0.05, compared with the control. (F) TUNEL assays show apoptotic cells in tumors from mice treated with vehicle control or I3C. Note the presence of only few apoptotic cells in tumors from the control mice (top panel), compared with the abundant apoptotic cells in tumors from the I3C-treated mice (bottom panel). Magnification, × 100.

Finally, we examined the effects of I3C against ATLL *in vivo*. SCID mice (*n *= 12) were inoculated with HUT-102 and then divided into two groups: untreated mice (*n *= 6) and I3C-treated mice (*n *= 6). Treatment commenced on the day after inoculation and the effects of treatment on tumorigenicity were assessed over four weeks. During treatment, all mice displayed no adverse events with respect to general appearance, body weight (Figure 7A) and food intake. I3C did not affect tumor incidence but significantly slowed the growth of the transplanted tumors (Figure 7B). After 28-day treatment, I3C significantly decreased tumor volume compared with vehicle-treated mice (*P *< 0.05) (Figure 7C). Statistically similar differences were found in tumor weights at necropsy (*P *< 0.05, Figure 7D and 7E). Terminal deoxynucleotidyl transferase mediated nick labeling (TUNEL) assay showed few apoptotic cells in tumors from untreated mice, while abundant apoptotic cells were noted in tumors from I3C-treated mice (Figure 7F). At necropsy, gross and histopathological examinations showed no apparent pathological findings, neoplastic lesions or metastatic tumors in the lungs, liver, pancreas, kidneys, spleen or large bowel in all mice. These results suggest that I3C is therapeutically beneficial in mice with ATLL.

## Discussion

In contrast to the latest progress in understanding HTLV-1 infection, pathogenesis and mode of action, more progress in developing therapies for these infected cells is needed. There has been only very limited improvement in the prognosis of ATLL during the past several years. However, few well established pathways including NF-κB and AP-1 have been shown to be tightly regulated in HTLV-1-infected T-cells and therefore providing viable targets for treatment [7,26].

The present investigation shows that I3C selectively inhibits cell viability of HTLV-1-infected T-cell lines and primary ATLL cells. The 50% inhibitory drug concentration (IC50) values in HTLV-1-infected T-cell lines and primary ATLL cells were 23.7–67.4 μM and 18.3–69.5 μM, respectively. We also showed that I3C induces G_1_/S cell-cycle arrest and apoptosis of HTLV-1-infected T-cell lines. In addition, our results provide mechanistic information on how I3C exerts its cytostatic and proapoptotic effects on HTLV-1-infected T-cell lines (i.e., by inhibiting NF-κB and AP-1 activity). Inhibition of NF-κB activity is mediated by blocking the phosphorylation of the NF-κB inhibitory protein IκBα and by preventing nuclear translocation of the NF-κB complex. Suppression of constitutive NF-κB activation by I3C in HTLV-1-infected T-cell lines is consistent with previous reports, which showed the suppression of constitutive NF-κB in PC3 prostate cancer cells, MDA-MB-231 breast cancer cells and acute myelogenous leukemia cells [37-39]. Our studies are the first to indicate that I3C also inhibits JunD expression, resulting in the suppression of AP-1 DNA-binding.

We showed that I3C inhibited NF-κB-regulated gene products involved in cell proliferation (*e.g*., cyclin D1, cyclin D2, Cdk4 and Cdk6) and antiapoptosis (*e.g*., XIAP, survivin and Bcl-2). Although cyclin D1 expression is regulated by NF-κB [27], AP-1 proteins also bind directly to the cyclin D1 promoter and activate it [40]. Furthermore, the cyclin D2 promoter contains NF-κB and AP-1 sites [41]. It is therefore likely that NF-κB and AP-1, in concert, support proliferation of HTLV-1-infected T cells by activating cyclin D1 and cyclin D2. We speculate that I3C inhibits cyclin D1 and cyclin D2 expression through the suppression of both NF-κB and AP-1, resulting in the induction of cell-cycle arrest at the G_1 _phase. Although Bcl-xL expression is regulated by NF-κB and AP-1, STAT and Ets also regulate its expression [42]. Therefore, I3C might fail to affect the expression of Bcl-xL.

The HTLV-1 encodes the oncoptotein Tax from its *pX *gene and the minus strand of the provirus encodes HBZ. Tax and HBZ play a central role in leukemogenesis of ATLL [5]. Tax activates not only viral replication but also induces the expression of cellular genes through NF-κB and AP-1 activation. HBZ interacts with JunD to activate the transcription of JunD-dependent promoters of cellular genes [43,44]. HBZ RNA promotes T-cell proliferation and upregulates E2F1 transcription [45]. We examined the levels of Tax and HBZ expression in HTLV-1-infected T-cell lines, HUT-102 and TL-OmI, but both were not molecular targets of I3C treatment.

Increasing attention is being paid to the possible use of agents that prevent the development of ATLL in individuals at high risk. For this purpose, the use of natural compounds for ATLL prevention has practical advantages with regard to availability, suitability for oral application, regulatory approval and mechanisms of action. Candidate substances present in foods and plants have been identified by experimental studies. Green tea polyphenols inhibit *in vitro *growth of ATLL cells as well as an HTLV-1-infected T-cell line, by inducing apoptosis [46]. The daily intake of capsulated green tea for 5 months significantly diminished the HTLV-1 provirus load compared with the controls [47].

Given the pharmacological safety of I3C (established through centuries of dietary intake), our study suggests that this compound has great potential as both a chemopreventive and a chemotherapeutic agent, especially when used in combination with other agents. Whether the concentrations of I3C used in our studies are achievable *in vivo *remains to be determined. After oral administration of I3C (250 mg/kg) to mice, the compound was rapidly absorbed and reached a peak concentration of 4.1 μg/ml at the earliest sampling time point of 15 min after dose [48]. I3C was rapidly and extensively distributed into sampled tissues, with highest concentrations in the liver (24.5 μg/g tissue). This concentration is equivalent to 165.4 μM, suggesting that the therapeutically effective concentrations of I3C used in the present studies may be achievable *in vivo*.

ATLL remains of poor prognosis. Novel effective drugs are warranted to reduce the emergence of resistant clones. Because the average Japanese ATLL patient is 60 year old, severe side effects and complications such as serious infections due to anti-cancer drugs are major problems in the clinical setting. Natural compounds may be safer than the currently available chemotherapeutic drugs. In particular, I3C might be useful in elderly patients or in immunocompromised patients because of its safety and lack of known toxicity. Overall, our results demonstrate that I3C is a potent inhibitor of NF-κB and AP-1 activation, which may explain its antiproliferative and proapoptotic effects. Our results further extend the potential preventive and therapeutic application of I3C for ATLL.

## Conclusion

We have demonstrated that a dietary agent, I3C, has potent antiproliferative and proapoptotic properties *in vitro *and is systemically active against aggressive ATLL in mice. In addition, I3C is well tolerated by the host animal in therapeutically beneficial doses, making it an attractive candidate for further preclinical testing as an anti-ATLL agent.

## Methods

### Cells

HTLV-1-infected T-cell lines, HUT-102 [1], MT-4 [49] and TL-OmI [50], HTLV-1-uninfected T-cell line, Jurkat, MOLT-4 and CCRF-CEM, were cultured in RPMI 1640 medium supplemented with 10% heat-inactivated fetal bovine serum (JRH Biosciences, Lenexa, KS), 50 U/ml penicillin and 50 μg/ml streptomycin. HUT-102 and MT-4 are HTLV-1-transformed T-cell lines and constitutively express viral genes including Tax. TL-OmI is a T-cell line of leukemic cell origin that was established from a patient with ATLL and does not express viral genes. Previously untreated ATLL patients were investigated. PBMC were isolated from four healthy volunteers and eight patients with acute type ATLL, using Ficoll-Paque density gradient centrifugation (GE Healthcare Biosciences, Uppsala, Sweden). All samples were obtained after informed consent. The diagnosis of ATLL was based on clinical features, hematological findings and the presence of anti-HTLV-1 antibodies in the sera. Monoclonal HTLV-1 provirus integration into the DNA of leukemic cells was confirmed by Southern blot hybridization in all patients (data not shown).

### Reagents

I3C was purchased from Calbiochem (La Jolla, CA). A 200 mM solution was prepared in dimethyl sulfoxide (DMSO), stored as small aliquots at -80°C, and then thawed and diluted as needed in cell culture medium. Control cultures received the same concentration of DMSO (0.05%), similar to those used for the experimental cultures. Rabbit polyclonal antibodies to cyclin D2, survivin, IκBα and JunD were purchased from Santa Cruz Biotechnology (Santa Cruz, CA). Rabbit polyclonal antibody to Bcl-xL was purchased from BD Transduction Laboratories (San Jose, CA). Mouse monoclonal antibodies to Bcl-2, Bax, Cdk4, Cdk6 and actin were purchased from NeoMarkers (Fremont, CA). Mouse monoclonal antibodies to XIAP and cyclin D1 were purchased from Medical & Biological Laboratories (MBL; Nagoya, Japan). Mouse monoclonal antibody to phospho-IκBα (Ser32/36) and rabbit polyclonal antibody to cleaved PARP and Bak were purchased from Cell Signaling Technology (Beverly, MA). Antibody to Tax, Lt-4, was described previously [51].

### Cell viability and apoptosis assays

The effect of I3C on cell viability was examined by the cell proliferation reagent, WST-8 (Wako Pure Chemical Industries, Osaka, Japan). Briefly, 1 × 10^5 ^cells/ml (cell lines) or 1 × 10^6 ^cells/ml (PBMC) were incubated in a 96-well microculture plate in the absence or presence of various concentrations of I3C. After 72 h (cell lines) or 24 h (PBMC) of culture, WST-8 (5 μl) was added for the last 4 h of incubation and absorbance at 450 nm was measured using an automated microplate reader. Measurement of mitochondrial dehydrogenase cleavage of WST-8 to formazan dye provides an indication of the level of cell viability. Apoptotic events in cells were detected by staining with phycoerythrin-conjugated APO2.7 monoclonal antibody (Beckman Coulter, Marseille, France) [52] and analyzed by flow cytometry (Epics XL, Beckman Coulter, Fullerton, CA). For analysis of morphologic changes of nuclei, cells were stained by 10 μg/ml Hoechst 33342 (Wako Pure Chemical Industries) and photographed through an ultraviolet filter using Olympus IX70 microscope (Olympus, Tokyo, Japan).

### Cell-cycle analysis

Cell-cycle analysis was performed with the CycleTEST PLUS DNA reagent kit (Becton Dickinson Immunocytometry Systems, San Jose, CA). In brief, 1 × 10^6 ^cells were washed with a buffer solution containing sodium citrate, sucrose and DMSO, suspended in a solution containing RNase A and stained with 125 μg/ml propidium iodide for 10 min. After passing the cells through a nylon mesh, cell suspensions were analyzed on an Epics XL. The distribution of cell-cycle phases with different DNA contents was determined.

### In vitro measurement of caspase activity

Cell extracts were recovered with the use of the cell lysis buffer and assessed for caspase-3, -8 and -9 activities using colorimetric probes (MBL). The colorimetric caspase assay kits are based on detection of the chromophore ρ-nitroanilide after cleavage from caspase-specific-labeled substrates. Colorimetric readings were performed in an automated microplate reader at an optical density of 400 nm.

### Western blot analysis

Cells were lysed in a buffer containing 62.5 mM Tris-HCl (pH 6.8), 2% SDS, 10% glycerol, 6% 2-mercaptoethanol and 0.01% bromophenol blue. Samples were subjected to electrophoresis on SDS-polyacrylamide gels followed by transfer to a polyvinylidene difluoride membrane and probing with the specific antibodies. The bands were visualized with the enhanced chemiluminescence kit (GE Healthcare Unlimited, Buckinghamshire, UK).

### RT-PCR

Total RNA was extracted with Trizol (Invitrogen, Carlsbad, CA) according to the protocol provided by the manufacturer. First-strand cDNA was synthesized from 5 μg total cellular RNA using an RNA PCR kit (Takara Bio, Otsu, Japan) with random primers. Thereafter, cDNA was amplified for 40 cycles for Tax, 35 cycles for HBZ and 28 cycles for β-actin. The sequences of the primers were as follows: for Tax, sense, 5'-CCGGCGCTGCTCTCATCCCGGT-3' and antisense, 5'-GGCCGAACATAGTCCCCCAGAG-3'; for HBZ, sense, 5'-CCGGCGCTGCTCTCATCCCGGT-3' and antisense, 5'-GGCCGAACATAGTCCCCCAGAG-3'; and for β-actin, sense, 5'-GTGGGGCGCCCCAGGCACCA-3' and antisense, 5'-CTCCTTAATGTCACGCACGATTTC-3'. Cycling conditions were as follows: denaturing at 94°C for 30 s, annealing at 60°C for 30 s, and extension at 72°C for 30 sec (for Tax and HBZ) or for 90 s (for β-actin). The PCR products were fractionated on 2% agarose gels and visualized by ethidium bromide staining.

### Detection of NF-κB p65

Cells were cultured with or without I3C for 12 h, and then fixed with paraformaldehyde for 10 min. For NF-κB p65 staining, the cells were permeabilized with 0.1% saponin in phosphate-buffered saline containing 1% bovine serum albumin. The cells were then incubated with a fluorescein isothiocyanate-conjugated rabbit polyclonal antibody for NF-κB p65 (Santa Cruz Biotechnology) for 45 min at 4°C. Protein localization was detected using fluorescence microscopy (Olympus).

### Preparation of nuclear extracts and EMSA

Cells were cultured and examined for inhibition of NF-κB and AP-1 after exposure to I3C for 12 h. Nuclear proteins were extracted, and NF-κB and AP-1 binding activities to NF-κB and AP-1 elements were examined by EMSA as described previously [25,35]. In brief, 5 μg of nuclear extracts were preincubated in a binding buffer containing 1 μg poly-deoxy-inosinic-deoxy-cytidylic acid (GE Healthcare Biosciences), followed by the addition of ^32^P-labeled oligonucleotide probe containing NF-κB and AP-1 elements (approximately 50,000 cpm). These mixtures were incubated for 15 min at room temperature. The DNA protein complexes were separated on 4% polyacrylamide gels and visualized by autoradiography. The probes used were prepared by annealing the sense and antisense synthetic oligonucleotides; a typical NF-κB element from the interleukin-2 receptor α chain gene (5'-gatcCGGCAGGGGAATCTCCCTCTC-3') and an AP-1 element of the interleukin-8 gene (5'-gatcGTGATGACTCAGGTT-3'). The oligonucleotide 5'-gatcTGTCGAATGCAAATCACTAGAA-3', containing the consensus sequence of the octamer binding motif, was used to identify specific binding of the transcription factor Oct-1. This transcription factor regulates the transcription of a number of so-called housekeeping genes. The above underlined sequences represent the NF-κB, AP-1 or Oct-1 binding site.

### In vivo administration of I3C

Five-week-old female C.B-17/Icr-SCID mice obtained from Ryukyu Biotec (Urasoe, Japan) were maintained in containment level 2 cabinets and provided with autoclaved food and water *ad libitum*. Mice were engrafted with 1 × 10^7 ^HUT-102 cells by subcutaneous injection in the postauricular region and randomly placed into two cohorts of six mice each that received vehicle or I3C. Treatment was initiated on the day after cell injection. I3C was dissolved in soybean oil at a concentration of 3.3 mg/ml, and 50 mg/kg body weight of I3C was administered by oral gavage every day for 28 days. Control mice received the same volume of the vehicle (soybean oil) only. Body weight and tumor numbers and size were monitored once a week. All mice were sacrificed on day 28, and then the tumors were dissected out and their weight was physically measured. Thereafter, tumors were fixed for paraffin embedding and tissue sectioning. Analysis of DNA fragmentation by fluorescent TUNEL was performed using a commercial kit (Takara Bio) as described in the instructions provided by the manufacturer. This experiment was performed according to the Guidelines for the Animal Experimentation of the University of the Ryukyus and was approved by the Animal Care and Use Committee of the same University.

### Statistical analysis

Data were expressed as mean ± SD. Mann-Whitney's *U*-test and Student's *t*-test were used, as appropriate. A *P *value less than 0.05 denoted the presence of statistical significance.

## Competing interests

The authors declare that they have no competing interests.

## Authors' contributions

YM contributed to the design of the study, evaluated the data, drafted the manuscript and performed all experimental procedures except as noted. SS carried out the TUNEL assay. CI performed the therapeutic intervention in SCID mouse model. YT provided the antibody to Tax. TO, JU, NT and KO provided study materials. NM conceived the study, contributed to the design and coordination of the experiments, and critically reviewed and edited the manuscript. All authors read and approved the final manuscript.

## References

[B1] Poiesz BJ, Ruscetti FW, Gazdar AF, Bunn PA, Minna JD, Gallo RC (1980). Detection and isolation of type C retrovirus particles from fresh and cultured lymphocytes of a patient with cutaneous T-cell lymphoma. Proc Natl Acad Sci USA.

[B2] Hinuma Y, Nagata K, Hanaoka M, Nakai M, Matsumoto T, Kinoshita K, Shirakawa S, Miyoshi I (1981). Adult T-cell leukemia: antigen in an ATL cell line and detection of antibodies to the antigen in human sera. Proc Natl Acad Sci USA.

[B3] Yoshida M, Miyoshi I, Hinuma Y (1982). Isolation and characterization of retrovirus from cell lines of human adult T-cell leukemia and its implication in the disease. Proc Natl Acad Sci USA.

[B4] Yamada Y, Tomonaga M, Fukuda H, Hanada S, Utsunomiya A, Tara M, Sano M, Ikeda S, Takatsuki K, Kozuru M, Araki K, Kawano F, Niimi M, Tobinai K, Hotta T, Shimoyama M (2001). A new G-CSF-supported combination chemotherapy, LSG15, for adult T-cell leukaemia-lymphoma: Japan Clinical Oncology Group Study 9303. Br J Haematol.

[B5] Matsuoka M, Jeang K-T (2007). Human T-cell leukaemia virus type 1 (HTLV-1) infectivity and cellular transformation. Nat Rev Cancer.

[B6] Grassmann R, Aboud M, Jeang K-T (2005). Molecular mechanisms of cellular transformation by HTLV-1 Tax. Oncogene.

[B7] Hall WW, Fujii M (2005). Deregulation of cell-signaling pathways in HTLV-1 infection. Oncogene.

[B8] Boxus M, Twizere J-C, Legros S, Dewulf J-F, Kettmann R, Willems L (2008). The HTLV-I Tax interactome. Retrovirology.

[B9] Loub WD, Wattenberg LW, Davis DW (1975). Aryl hydrocarbon hydroxylase induction in rat tissues by naturally occurring indoles of cruciferous plants. J Natl Cancer Inst.

[B10] Wattenberg LW, Loub WD (1978). Inhibition of polycyclic aromatic hydrocarbon-induced neoplasia by naturally occurring indoles. Cancer Res.

[B11] He Y-H, Friesen MD, Ruch RJ, Schut HAJ (2000). Indole-3-carbinol as a chemopreventive agent in 2-amino-1-methyl-6-phenylimidazo[4,5-*b*]pyridine (PhIP) carcinogenesis: inhibition of PhIP-DNA adduct formation, acceleration of PhIP metabolism, and induction of cytochrome P450 in female F344 rats. Food Chem Toxicol.

[B12] Jin L, Qi M, Chen D-Z, Anderson A, Yang G-Y, Arbeit JM, Auborn KJ (1999). Indole-3-carbinol prevents cervical cancer in human papilloma virus type 16 (HPV16) transgenic mice. Cancer Res.

[B13] Oganesian A, Hendricks JD, Williams DE (1997). Long term dietary indole-3-carbinol inhibits diethylnitrosamine-initiated hepatocarcinogenesis in the infant mouse model. Cancer Lett.

[B14] Bell MC, Crowley-Nowick P, Bradlow HL, Sepkovic DW, Schmidt-Grimminger D, Howell P, Mayeaux EJ, Tucker A, Turbat-Herrera EA, Mathis JM (2000). Placebo-controlled trial of indole-3-carbinol in the treatment of CIN. Gynecol Oncol.

[B15] Reed GA, Peterson KS, Smith HJ, Gray JC, Sullivan DK, Mayo MS, Crowell JA, Hurwitz A (2005). A phase I study of indole-3-carbinol in women: tolerability and effects. Cancer Epidemiol Biomarkers Prev.

[B16] Reed GA, Arneson DW, Putnam WC, Smith HJ, Gray JC, Sullivan DK, Mayo MS, Crowell JA, Hurwitz A (2006). Single-dose and multiple-dose administration of indole-3-carbinol to women: pharmacokinetics based on 3,3'-diindolylmethane. Cancer Epidemiol Biomarkers Prev.

[B17] Naik R, Nixon S, Lopes A, Godfrey K, Hatem MH, Monaghan JM (2006). A randomized phase II trial of indole-3-carbinol in the treatment of vulvar intraepithelial neoplasia. Int J Gynecol Cancer.

[B18] Sarkar FH, Li Y (2004). Indole-3-carbinol and prostate cancer. J Nutr.

[B19] Manson MM (2005). Inhibition of survival signaling by dietary polyphenols and indole-3-carbinol. Eur J Cancer.

[B20] Kim YS, Milner JA (2005). Targets for indole-3-carbinol in cancer prevention. J Nutr Biochem.

[B21] Aggarwal BB, Ichikawa H (2005). Molecular targets and anticancer potential of indole-3-carbinol and its derivatives. Cell Cycle.

[B22] Howells LM, Hudson EA, Manson MM (2005). Inhibition of phosphatidylinositol 3-kinase/protein kinase B signaling is not sufficient to account for indole-3-carbinol-induced apoptosis in some breast and prostate tumor cells. Clin Cancer Res.

[B23] Mesnard J-M, Barbeau B, Devaux C (2006). HBZ, a new important player in the mystery of adult T-cell leukemia. Blood.

[B24] Naugler WE, Karin M (2008). NF-κB and cancer-identifying targets and mechanisms. Curr Opin Genet Dev.

[B25] Mori N, Fujii M, Ikeda S, Yamada Y, Tomonaga M, Ballard DW, Yamamoto N (1999). Constitutive activation of NF-κB in primary adult T-cell leukemia cells. Blood.

[B26] Sun S-C, Yamaoka S (2005). Activation of NF-κB by HTLV-I and implications for cell transformation. Oncogene.

[B27] Hinz M, Krappmann D, Eichten A, Heder A, Scheidereit C, Strauss M (1999). NF-κB function in growth control: regulation of cyclin D1 expression and G_0_/G_1_-to-S-phase transition. Mol Cell Biol.

[B28] Huang Y, Ohtani K, Iwanaga R, Matsumura Y, Nakamura M (2001). Direct trans-activation of the human cyclin D2 gene by the oncogene product Tax of human T-cell leukemia virus type I. Oncogene.

[B29] Iwanaga R, Ohtani K, Hayashi T, Nakamura M (2001). Molecular mechanism of cell cycle progression induced by the oncogene product Tax of human T-cell leukemia virus type I. Oncogene.

[B30] Grossmann M, O'Reilly LA, Gugasyan R, Strasser A, Adams JM, Gerondakis S (2000). The anti-apoptotic activities of Rel and RelA required during B-cell maturation involve the regulation of Bcl-2 expression. EMBO J.

[B31] Stehlik C, de Martin R, Kumabashiri I, Schmid JA, Binder BR, Lipp J (1998). Nuclear factor (NF)-κB-regulated X-chromosome-linked *iap *gene expression protects endothelial cells from tumor necrosis factor α-induced apoptosis. J Exp Med.

[B32] Kawakami H, Tomita M, Matsuda T, Ohta T, Tanaka Y, Fujii M, Hatano M, Tokuhisa T, Mori N (2005). Transcriptional activation of survivin through the NF-κB pathway by human T-cell leukemia virus type I Tax. Int J Cancer.

[B33] Nakazato T, Okudaira T, Ishikawa C, Nakama S, Sawada S, Tomita M, Uchihara J, Taira N, Masuda M, Tanaka Y, Ohshiro K, Takasu N, Mori N (2008). Anti-adult T-cell leukemia effects of a novel synthetic retinoid, Am80 (Tamibarotene). Cancer Sci.

[B34] Chen ZJ, Parent L, Maniatis T (1996). Site-specific phosphorylation of IκBα by a novel ubiquitination-dependent protein kinase activity. Cell.

[B35] Mori N, Fujii M, Iwai K, Ikeda S, Yamasaki Y, Hata T, Yamada Y, Tanaka Y, Tomonaga M, Yamamoto N (2000). Constitutive activation of transcription factor AP-1 in primary adult T-cell leukemia cells. Blood.

[B36] Iwai K, Mori N, Oie M, Yamamoto N, Fujii M (2001). Human T-cell leukemia virus type 1 tax protein activates transcription through AP-1 site by inducing DNA binding activity in T cells. Virology.

[B37] Chinni SR, Li Y, Upadhyay S, Koppolu PK, Sarkar FH (2001). Indole-3-carbinol (I3C) induced cell growth inhibition, G1 cell cycle arrest and apoptosis in prostate cancer cells. Oncogene.

[B38] Rahman KMW, Sarkar FH, Banerjee S, Wang Z, Liao DJ, Hong X, Sarkar NH (2006). Therapeutic intervention of experimental breast cancer bone metastasis by indole-3-carbinol in SCID-human mouse mode. Mol Cancer Ther.

[B39] Takada Y, Andreeff M, Aggarwal BB (2005). Indole-3-carbinol suppresses NF-κB and IκBα kinase activation, causing inhibition of expression of NF-κB-regulated antiapoptotic and metastatic gene products and enhancement of apoptosis in myeloid and leukemia cells. Blood.

[B40] Shaulian E, Karin M (2001). AP-1 in cell proliferation and survival. Oncogene.

[B41] Brooks AR, Shiffman D, Chan CS, Brooks EE, Milner PG (1996). Functional analysis of the human cyclin D2 and cyclin D3 promoters. J Biol Chem.

[B42] Sevilla L, Zaldumbide A, Pognonec P, Boulukos KE (2001). Transcriptional regulation of the bcl-x gene encoding the anti-apoptotic Bcl-xL protein by Ets, Rel/NF-κB, STAT and AP1 transcription factor families. Histol Histopathol.

[B43] Thébault S, Basbous J, Hivin P, Devaux C, Mesnard J-M (2004). HBZ interacts with JunD and stimulates its transcriptional activity. FEBS Lett.

[B44] Kuhlmann A-S, Villaudy J, Gazzolo L, Castellazzi M, Mesnard J-M, Duc Dodon M (2007). HTLV-1 HBZ cooperates with JunD to enhance transcription of the human telomerase reverse transcriptase gene (hTERT). Retrovirology.

[B45] Satou Y, Yasunaga J, Yoshida M, Matsuoka M (2006). *HTLV-I basic leucine zipper factor *gene mRNA supports proliferation of adult T cell leukemia cells. Proc Natl Acad Sci USA.

[B46] Li H-C, Yashiki S, Sonoda J, Lou H, Ghosh SK, Byrnes JJ, Lema C, Fujiyoshi T, Karasuyama M, Sonoda S (2000). Green tea polyphenols induce apoptosis *in vitro *in peripheral blood T lymphocytes of adult T-cell leukemia patients. Jpn J Cancer Res.

[B47] Sonoda J, Koriyama C, Yamamoto S, Kozako T, Li HC, Lema C, Yashiki S, Fujiyoshi T, Yoshinaga M, Nagata Y, Akiba S, Takezaki T, Yamada K, Sonoda S (2004). HTLV-1 provirus load in peripheral blood lymphocytes of HTLV-1 carriers is diminished by green tea drinking. Cancer Sci.

[B48] Anderton MJ, Manson MM, Verschoyle RD, Gescher A, Lamb JH, Farmer PB, Steward WP, Williams ML (2004). Pharmacokinetics tissue disposition of indole-3-carbinol and its acid condensation products after oral administration to mice. Clin Cancer Res.

[B49] Yamamoto N, Okada M, Koyanagi Y, Kannagi M, Hinuma Y (1982). Transformation of human leukocytes by cocultivation with an adult T cell leukemia virus producer cell line. Science.

[B50] Sugamura K, Fujii M, Kannagi M, Sakitani M, Takeuchi M, Hinuma Y (1984). Cell surface phenotypes and expression of viral antigens of various human cell lines carrying human T-cell leukemia virus. Int J Cancer.

[B51] Tanaka Y, Yoshida A, Takayama Y, Tsujimoto H, Tsujimoto A, Hayami M, Tozawa H (1990). Heterogeneity of antigen molecules recognized by anti-tax1 monoclonal antibody Lt-4 in cell lines bearing human T cell leukemia virus type I and related retroviruses. Jpn J Cancer Res.

[B52] Zhang C, Ao Z, Seth A, Schlossman SF (1996). A mitochondrial membrane protein defined by a novel monoclonal antibody is preferentially detected in apoptotic cells. J Immunol.

